# Assessing Enamel Thickness to Estimate Interproximal Reduction: A CBCT‐Based Study

**DOI:** 10.1002/cre2.70083

**Published:** 2025-02-17

**Authors:** Enrique González‐García, Nasib Balut‐Chahin, Claudia Daniela Rojo‐Arce, María Eugenia Jiménez Corona, Luis Pablo Cruz‐Hervert, Jean Marc Retrouvey

**Affiliations:** ^1^ Department of Orthodontics Technological University of Mexico Mexico City Mexico; ^2^ Department of Orthodontics University of Valle Cali Colombia; ^3^ Department of Orthodontics University of Baja California Mexicali Baja California Mexico; ^4^ Posgraduate and Research Studies Division, Dentistry Faculty National Autonomous University of Mexico México City Mexico; ^5^ Postgraduate and Research Studies Division, Dentistry Faculty National Autonomous University of Mexico Mexico City Mexico; ^6^ Department of Epidemiology Institute of Cardiology Ignacio Chavez Mexico City Mexico; ^7^ Baylor College of Medicine Houston Texas USA

**Keywords:** CBCT, enamel availability, enamel thickness, interproximal reduction, IPR, PETa, PETr, proximal enamel thickening

## Abstract

**Objectives:**

The aims of this study were to (1) estimate the mesial and distal proximal enamel thickness available (PETa), (2) estimate the proximal enamel thickness remaining (PETr) on the basis of planned IPR, and (3) assess PETr‐associated risks with varying IPR amounts.

**Materials and Methods:**

A cross‐sectional study was conducted using CBCT scans. PETa was estimated using on‐demand software. Mesial and distal PET were measured at the middle third of the crown. The means and 95% confidence intervals (CIs) of the PETa and PETr data are reported. Differences between the mesial and distal PETa values were compared.

**Results:**

A total of 1615 teeth were analyzed via CBCT. The mean PETa values ranged from 0.96 to 1.29 mm (mesial) and from 0.98 to 1.25 mm (distal). Differences between mesial and distal PETa were statistically significant, averaging 0.10 mm proximally (*p* < 0.050). In particular, these differences were observed in cuspids, including tooth 13 (1.18 ± 0.24 vs. 1.25 ± 0.28; *p* = 0.021), tooth 23 (1.25 ± 0.26 vs. 1.15 ± 0.28; *p* < 0.001), tooth 33 (1.22 ± 0.26 vs. 1.10 ± 0.23; *p* < 0.001), and tooth 43 (1.29 ± 0.24 vs. 1.13 ± 0.20; *p* < 0.001). The mean PETr values for single‐site IPRs < 0.4 mm were 0.58 mm (mesial) and 0.57 mm (distal). Exceeding a single‐site IPR of 0.20 mm significantly increased the proportion of interproximal sites classified as moderate or high risk, particularly in teeth with thinner enamel (< 0.7 mm).

**Conclusions:**

PETa and PETr are critical for determining safe and individualized IPR. CBCT‐based PETa evaluations are strongly recommended when single‐site IPRs exceeding 0.20 mm are planned to increase precision and reduce the risk of excessive enamel reduction.

## Summary

- PETa mean values ranged from 0.96 to 1.29 mm for the mesial direction and from 0.98 to 1.25 mm for the distal direction.
- Differences between mesial and distal PETa were statistically but not clinically significant.
- The mean PETr for a single‐site IPR < 0.4 mm was 0.58 mm for mesial sites and 0.57 mm for distal sites.
- Exceeding a single‐site IPR (0.20 mm) will increase the proportion of sites at moderate or high risk.
- CBCT PETa evaluations are recommended for single‐site IPRs exceeding 0.20 mm or greater.

**Abbreviations**
CBCT: cone‐beam computed tomography
IPR: interproximal reduction
MPR: multiplanar reduction
PETa: proximal thickness available
PETr: proximal thickness remaining

## 1 Background

Interproximal reduction (IPR) was first introduced by Sheridan in 1944 and is now a widely used technique in orthodontic treatment to facilitate tooth movement and perform alignment (Sheridan 1987, 1997; Chudasama and Sheridan 2007; Ballard 1944). This procedure involves selectively reducing interproximal enamel thickness using diamond discs, burs, or lightning strips (Chudasama and Sheridan 2007; Sehgal et al. 2019; Pindoria, Fleming, and Sharma 2016; Kalemaj and Levrini 2020; Laganà et al. 2021; Johner et al. 2013; Felice et al. 2020; Danesh et al. 2007; Baysal, Uysal, and Usumez 2007). When performed correctly, IPR is a standard practice in orthodontics to address crowding, Bolton discrepancies, overjet, midline correction, and aesthetic concerns. Additionally, it provides a nonextraction alternative in specific cases when combined with other orthodontic strategies (Sheridan 1997; Chudasama and Sheridan 2007; Pindoria, Fleming, and Sharma 2016; Kalemaj and Levrini 2020; Laganà et al. 2021; Johner et al. 2013; Felice et al. 2020; Sarig et al. 2013; Radnzic 1988; d'Apuzzo et al. 2019).

Despite its widespread use, traditional IPR planning has focused primarily on creating the necessary space for alignment, often relying on the visible width of crowns without considering actual enamel thickness. This approach often lacks precision and may lead to excessive enamel reduction, compromising tooth structure and causing adverse outcomes. Accurate IPR planning is essential not only for achieving alignment but also for preserving enamel integrity. Overreduction of enamel can result in structural weakening, heightened sensitivity, and increased susceptibility to caries. Furthermore, uneven enamel distribution due to imprecise planning may negatively impact the long‐term prognosis of teeth, increasing periodontal risk and compromising stability.

To prevent these complications, individualized IPR planning based on proximal enamel thickness remaining (PETr) is critical. Factors such as tooth morphology, race, sex, and prior treatments influence PETr, highlighting the need for precision to avoid excessive enamel removal and achieve orthodontic goals. The recommended amount of enamel removal during IPR typically ranges between 0.20 and 0.50 mm per interproximal site (Sheridan 1987, 1997; Chudasama and Sheridan 2007; Paskow 1970; Rossouw and Tortorella 2003). However, these recommendations often fail to account for individual variations in PETr, which can differ on the basis of factors such as tooth type, morphology (Sarig et al. 2013; Radnzic 1988; Hall et al. 2007; Harris and Hicks 1998), race (Sarig et al. 2013; Vellini‐Ferreira et al. 2012), sex (Paknahad et al. 2022; Stroud, Buschang, and Goaz 1994), and prior dental or orthodontic treatments (d'Apuzzo et al. 2019; Paskow 1970; Chow et al. 2020; Barcoma et al. 2014). The personalization of IPR via proximal enamel thickness available (PETa) is essential for achieving the desired clinical outcomes while preserving enamel integrity (Paknahad et al. 2022; Sarig et al. 2015; Akli et al. 2020; Ei et al. 2019; Bian et al. 2020).

Numerous methods have been used to measure PETa, including bitewings, periapical radiography, microscopy, profilometry, and microcomputed tomography. However, these techniques present limitations, such as measurement errors caused by radiographic distortions, head tilting, or tooth positioning (Kim, Paik, and Lee 2007). Numerous methods have been used to measure PETa, including bitewings, periapical radiography, microscopy, profilometry, and microcomputed tomography. However, these techniques present limitations, such as measurement errors caused by radiographic distortions, head tilting, or tooth positioning (Olejniczak and Grine 2006; Smith et al. 2006; Daubert et al. 2016). Despite its potential, CBCT‐based PETa assessment remains underexplored, with few studies, such as those by Akli et al. (2020) and Bian et al. (2020), utilizing CBCT images for PETa estimation.

The optimal amount of enamel to be removed during an IPR procedure remains unclear, with a maximum IPR of 0.5 mm per interproximal site suggested. These suggestions appear to be made without previous evaluation, and there is a need to personalize the enamel for each tooth surface potentially involved. A meta‐analysis by Kailasam et al. (2021) reported mean enamel thicknesses ranging from 0.86 to 1.35 mm for the distal surface and from 0.76 to 1.38 mm for the mesial surfaces in 2021, with an overall mean difference of 0.1 mm (95% confidence interval (95% CI: 0.09–0.12 mm). The teeth with the greatest mean enamel thickness were the distal surfaces of the first upper molars (1.45 mm), whereas the lowest mean enamel thickness was recorded on the mesial surfaces of the lower central and lateral incisors (0.70 mm). Hudson (1956) was the first to propose that up to 50% enamel reduction could be safe, whereas Bosio (2022), who used the results of Kailasam et al. (2021) proposed a range of 0.42–0.72 mm for maxillary teeth and 0.35–0.76 mm for mandibular teeth per tooth, highlighting the variability in enamel thickness across tooth types and the need for individualized IPR planning.

Despite its widespread use, the optimal application of IPR remains unclear due to unresolved gaps in knowledge. Previous PETa studies often rely on outdated methods, with limited use of CBCT for accurate assessments. The existing IPR recommendations lack empirical validation and fail to consider enamel thickness variability, increasing the risk of overreduction. Additionally, insufficient data exist on remaining enamel thickness (PETr) after different levels of reduction, particularly for thin enamel areas prone to damage. Specifically, limited clarity exists regarding one‐ versus two‐sided IPR protocols and their implications for enamel preservation and tooth stability (Meredith et al. 2017). Although PETa measurement alone may not justify routine application, its use when clinically available can improve IPR precision.

Cone‐beam computed tomography (CBCT) represents a tool for assessing proximal enamel thickness (PETa) because of its ability to provide three‐dimensional imaging and accurate measurements of enamel and dentin structures. Unlike traditional radiographic methods, CBCT minimizes distortions caused by tooth positioning and head tilting, enabling precise visualization of enamel layers. Additionally, CBCT offers cross‐sectional views that eliminate overlapping structures, providing detailed and reliable data for treatment planning. These advantages make CBCT particularly valuable in cases where precise enamel evaluation is critical.

To address these gaps, this study aims to (1) estimate the overall PETa for the mesial and distal surfaces of types of teeth, (2) calculate the remaining proximal enamel thickness (PETr) on the basis of planned IPR values, and (3) assess the potential risks associated with IPR amounts ranging from 0.1 to 0.6 mm. The null hypothesis posited no significant PETa differences between mesial and distal surfaces and no teeth classified as high risk with IPR under 0.3 mm.

## 2 Methods

A retrospective cross‐sectional study analyzed 101 randomly selected CBCT records from 289 patient files via EPIDAT V.4.2. These records were obtained at a private practice from January to December 2020. CBCTs were obtained via a PLANMECA 2D (80–150 kHz, 50–84 kV, voxel size of 0.3 mm) in a private radiology center. Our inclusion criteria were as follows: (1) adult patients between 18 and 50 years of age at the time of CBCT recording to ensure full dental development and exclude significant age‐related enamel wear; (2) patients with no history of previous orthodontic treatment (e.g., braces, fixed, or removable retainers) to avoid alterations in enamel thickness caused by prior IPRs or tooth movement; and (3) patients with no evidence of craniofacial syndromes or conditions that could impact tooth morphology, enamel integrity or dental development, as assessed through clinical history and radiographic evaluation. Our exclusion criterion was the presence of image alterations in the CBCTs that impaired positive PETa identification.

**Figure 1:**
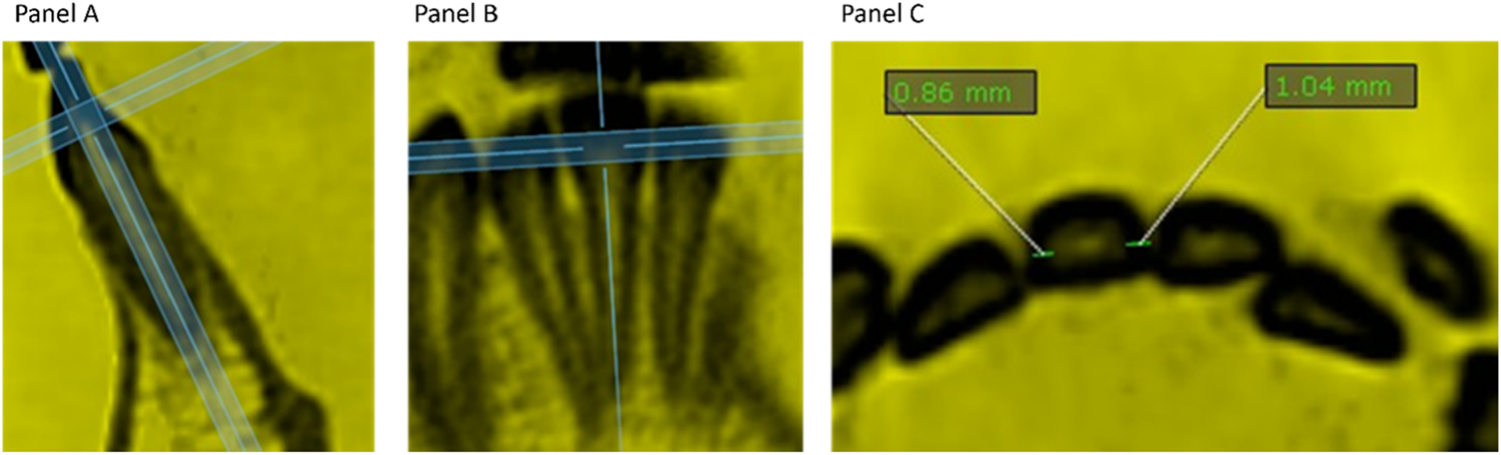
The proximal enamel thickness available (PETa) was measured using on‐demand software. Footnote: The provided images are segmented into three panels—Panel A, Panel B, and Panel C—each illustrating aspects of image analysis. Panel A and Panel B display cross‐sectional views with alignment guides, ensuring positioning for diagnostic evaluation. Panel C provides measurements of interproximal distances, marked at 0.86 mm and 1.04 mm. These measurements are integral for determining the suitability of interproximal reduction (IPR) in orthodontic treatment, balancing the need for space creation with the preservation of dental enamel integrity.

Proximal enamel thickness available (PETa) measurements were obtained via the OnDemand viewer. The process began with the selection of the 3D image (Figure 1A), which initiated the viewer opening. The “Restore” function was then selected to display the four critical views: sagittal, coronal, axial, and 3D.

The zoom was adjusted to reflect a 6 mm scale on the measurement sidebar. Once this view was set, image clarity was enhanced by setting the thickness (TH) to 2.0 for all sections. To improve the visibility of the enamel thickness, the “windowing” feature was utilized. From the toolbar at the bottom left, the “Blue” filter was selected from the list, followed by the “Invert” function. This process was performed to enhance and facilitate the differentiation between enamel and dentin. Importantly, this diagnostic approach was performed on the largest field of view (FOV) typically used for diagnosis in orthodontics, as the intention was to maximize the diagnostic benefits without the need for a second tomography. The marker was placed with precision in the axial, sagittal, and coronal directions to optimize the view of the tooth being measured, ensuring that it was positioned in the middle third in all views. The “enter” key was pressed to eliminate any excess lines, allowing for the measurement of enamel thickness using only the sidebar tool.

Axial slices were obtained from the left second premolar to the right second premolar in both dental arches, ensuring that the longitudinal axis of each tooth was centered in the three spatial planes. The enamel thickness was measured mesially and distally at the level of the middle third in the axial section, using the approach proposed by Sarig et al (2015). This area was selected because it is typically the interproximal contact zone. Although IPR can be performed directly at the contact point, measuring at the middle third provides a more representative assessment of the enamel thickness in this critical area of interest (Figure 1).

### 2.1 Variables

#### 2.1.1 Measurement of Proximal Enamel Thickness (PETa)

PETa was defined as the total distance in millimeters between the inner and outer enamel prior to IPR according to the coronal view in the middle third of the anatomical crown.

#### 2.1.2 PETr

PETr was defined as the remaining quantity of enamel thickness expressed in millimeters after theoretical subtraction of a one‐sided IPR of 0.1 mm (PETr‐10), 0.2 mm (PETr‐20), 0.3 mm (PETr‐30), 0.4 mm (PETr‐40), or 0.5 mm (PETr‐50).

#### 2.1.3 Threshold Values for PETr

The primary aim of this study was to evaluate the potential risks associated with PETr following IPR. This evaluation identifies thresholds to preserve enamel and minimize excessive reduction risks, as Sarig et al. (2013) suggested. Although no validated classification system currently exists for PETr, clinical thresholds can be inferred from concepts reported in the literature.

First, on the basis of Sheridan's recommendation (Sheridan 1987, 1997; Chudasama and Sheridan 2007) of a safe reduction limit of 0.25 mm and assuming an average PETa of 1 mm, an upper threshold for PETr was defined as ≥ 0.75 mm, representing no risk. Second, consistent with Hudson's proposal (Hudson 1956) that 50% PETa represents the maximum IPR limit and using the same mean PETa of 1 mm, a threshold range of ≥ 0.50 mm to < 0.75 mm was defined as low risk. Further thresholds were established on the basis of Stroud's findings (Stroud, Buschang, and Goaz 1994), which indicate that a minimum enamel thickness of 0.30 mm provides adequate protection for inner dental tissues. A range of ≥ 0.30 mm to < 0.50 mm was proposed to represent a moderate risk, whereas PETr values below 0.30 mm were considered a critical limit associated with a high risk for structural compromise.

As we previously described, no formal classification system exists, to our knowledge, for evaluating PETr. However, this approach aligns with routinely applied clinical findings reported in the literature and may serve as a practical reference for guiding IPR planning, balancing enamel preservation with orthodontic treatment outcomes.

### 2.2 PETa Data Measurements

PETa measurements in millimeters were performed by a single operator and previously standardized by an imaging specialist. PETa measurements were performed using a 3D multiplanar reconstruction (MPR) module from On‐Demand V 6.3.19045. The lights and contrasts were adjusted using the windowing button, and an inversion tool with a blue filter was used to optimize the definition of the PETa. The thickness of the slice was 2 mm, and a 6 mm zoom was used to improve the visualization of the PETa signal. A single operator cut coronal sections from the second left bicuspid to the second right bicuspid in both arches (maxillary and mandibular). Each slice was adjusted to the longitudinal axis for each tooth using the sagittal, coronal, and axial views. Mesial and distal PETa were measured at the height of the middle third of the radiographical crown using an axial view.

### 2.3 Reliability Assessment

To evaluate the reliability of the PETa measurement method, a total of 960 measurements were analyzed, comprising 480 at time 1 and 480 at time 2, obtained from 12 CBCT scans of 20 teeth, with measurements recorded mesially and distally.

Three statistical approaches were used to assess reliability: Dahlberg's formula, the intraclass correlation coefficient (ICC), and Bland‒Altman analysis. The measurement error calculated using Dahlberg's formula was 0.09 mm (±0.01 mm), indicating high precision and minimal measurement variability. The ICC, which estimates the correlation between individual measurements and the average measurements performed on the same target, demonstrated strong reliability, with an individual ICC value of 0.86 (95% CI: 0.71–0.942) and a mean ICC value of 0.92 (95% CI: 0.83–0.97), suggesting excellent consistency. The Bland‒Altman analysis revealed a bias of –0.02 mm (±0.13 mm), indicating a minimal systematic error, whereas Lin's concordance correlation coefficient for absolute agreement was 0.7980, with a *p* value of < 0.001, further confirming the strong agreement between the measurements. The results of these three approaches confirm that the PETa measurement method demonstrates a high level of reliability, establishing it as a robust tool for orthodontic measurement applications.

### 2.4 Sample Size Calculation

We performed a sample size calculation assuming a standard deviation of 0.5 mm, 95% confidence interval (95% CI), and precision of 0.1, and used a t distribution. We estimated a total sample size of 100 teeth to calculate a mean of 1 mm with a 95% CI and an accuracy of 0.1.

Additionally, we calculated that a minimum of 1135 sample sizes was needed for a multiple regression model with the following assumptions: (1) estimated effect size of 0.02, considered a low effect; (2) statistical power of 0.90; (3) 14 predictors, one for each tooth type, mesial/distal site, sex, and age; and (4) an alpha level of 0.05. Considering 100 measurements per mesial and distal site of each tooth, we will have approximately 4000 measurements, which is the minimum sample size needed.

### 2.5 Statistical Methods

We estimated the PETa and PETr population means and 95% CIs as well as the interquartile range, minimum, and maximum for each tooth (mesial and distal) in the total and small, medium, and large PETa categories. Additionally, for each tooth, we estimated the percentage above 0.5 mm of PETr (PETr%) and its 95% CI in total for small, medium, and large regions and the mean and SD of PETr after a one‐sided IPR of 0.10, 0.20, 0.50, 0.75 (PETr‐70), and 1 mm. Measurements of 0.1 mm may be biased due to the 0.3 mm tomographic slice, potentially compromising accuracy. However, including this measurement is necessary to identify risks, as a 0.1 mm IPR is minimal and commonly recommended during treatment.

### 2.6 Ethical Approval and Consent to Participate

This study was approved by the Research and Ethics Committee of the UNAM (CIE/0405/11/2021). The study utilized data from an image bank from a private radiological center, processed by a single employer with a signed confidential agreement contract, and no personal information was included; therefore, the images are entirely unidentifiable, there are no details on individuals reported within the manuscript, and consent for publication of images may not be needed.

## 3 Results

We evaluated 1615 teeth at the mesial and distal sites. The sample included 96 patient CBCT records, with 62.2% females (*n* = 51) and 37.8% males (*n* = 31). All patients were of Mexican descent. The ages ranged from 18 to 35 years, with a mean age of 24.8 years (standard deviation [S.D.] ±4.1).

### 3.1 Mesial and Distal PETa

The results of the mesial and distal PETa measurements, as well as their statistical comparisons for each tooth, are described in detail in Table 1. No consistent differences in enamel thickness have been observed between the mesial and distal sites of the same tooth. In cases where statistically significant differences have been detected, we deem these differences clinically irrelevant. To determine whether there was a substantial difference, we compared the PETa from the mesial and distal sites of the same tooth. The overall mean for each tooth ranged from 0.98 to 1.29 mm in the mesial plane and from 0.96 to 1.25 mm in the distal plane. The overall minimum thickness of PETa for all teeth was 0.1 mm for both the mesial and distal sites. For a total of 20 teeth, 13 (65%) mesial and 16 (80%) distal sites had PETa values equal to or less than 0.7 mm; in these cases, if a 0.25 mm single‐site IPR is performed, PETr may be 0.5 mm or less. Consistent mesial and distal differences were identified between the upper and lower cuspids as follows: tooth 13 (1.18 ± 0.24 vs. 1.25 ± 0.28; *p* = 0.021), tooth 23 (1.25 ± 0.26 vs. 1.15 ± 0.28; *p* < 0.001), tooth 33 (1.22 ± 0.26 vs. 1.10 ± 0.23; *p* < 0.001), and tooth 43 (1.29 ± 0.24 vs. 1.13 ± 0.20; *p* < 0.001). Differences between mesial and distal PETa ranged from 0.05 to 0.11 mm and were statistically significant (*p* < 0.050). Although statistically significant, these differences have no substantial clinical implications, as the variations are unlikely to affect clinical decisions (Table 1). Nevertheless, both teeth with significant differences and those without significant differences should be evaluated conservatively when assessing PETa, adopting an approach that assumes that mesial and distal differences exist to ensure safe and precise clinical outcomes (Table 1).

**Table 1:** Proximal enamel thickness available before interproximal enamel reduction.

Proximal enamel thickness available	n	Mesial							Distal							
		Mean	S.D.	Median	25th	75th	Min.	Max.	Mean	S.D.	Median	25th	75th	Min.	Max.	*p*‐value
Upper right second bicuspid	93	1.08	0.17	1.10	1	1.2	0.7	1.4	1.1	0.25	1.00	0.9	1.2	0.4	1.9	0.448
Upper right first bicuspid	95	1.05	0.19	1.00	0.9	1.2	0.7	1.4	0.99	0.27	1.00	0.9	1.2	0.1	1.6	0.021
Upper right cuspid	96	1.18	0.24	1.20	1	1.35	0.4	1.7	1.25	0.28	1.20	1	1.4	0.8	1.8	0.013
Upper right lateral incisor	95	1.07	0.24	1.00	0.9	1.2	0.7	1.6	1.05	0.25	1.00	0.9	1.2	0.1	1.7	0.266
Upper right central incisor	94	1.15	0.22	1.10	1	1.3	0.8	1.8	1.11	0.26	1.10	1	1.2	0.6	1.8	0.225
Upper left central incisor	93	1.17	0.24	1.10	1	1.3	0.7	2	1.18	0.32	1.10	1	1.2	0.1	1.9	0.789
Upper left lateral incisor	93	1.06	0.21	1.10	0.9	1.2	0.7	1.7	1.01	0.3	1.00	0.9	1.2	0.1	1.6	0.125
Upper left cuspid	94	1.25	0.26	1.20	1	1.4	0.8	1.7	1.15	0.28	1.10	1	1.3	0.4	1.7	< 0.001
Upper left first bicuspid	96	1.06	0.18	1.05	0.9	1.2	0.8	1.9	1.04	0.22	1.00	0.9	1.2	0.7	1.6	0.317
Upper left second bicuspid	91	1.02	0.14	1.00	0.9	1.1	0.8	1.3	1.07	0.2	1.00	1	1.1	0.8	1.8	0.023
Lower left second bicuspid	93	1.10	0.2	1.10	1	1.2	0.8	1.9	1.07	0.22	1.00	0.9	1.2	0.7	1.5	0.125
Lower left first bicuspid	96	1.08	0.18	1.00	0.9	1.2	0.8	1.6	1.08	0.18	1.00	1	1.2	0.8	1.7	0.79
Lower left cuspid	96	1.22	0.26	1.20	1	1.4	0.8	1.9	1.1	0.23	1.10	1	1.2	0.1	1.7	< 0.001
Lower left lateral incisor	96	0.99	0.16	1.00	0.9	1.1	0.7	1.5	1.01	0.23	1.00	0.9	1.2	0.1	1.8	0.437
Lower left central incisor	96	0.98	0.14	1.00	0.9	1.1	0.7	1.6	1.04	0.21	1.10	0.9	1.2	0.1	1.8	0.019
Lower right central incisor	96	1.05	0.23	1.00	0.9	1.2	0.1	2	0.99	0.24	0.90	0.9	1.1	0.4	1.8	0.015
Lower right lateral incisor	96	1.04	0.21	1.00	0.9	1.1	0.7	1.7	0.96	0.21	0.90	0.8	1	0.4	1.8	< 0.001
Lower right cuspid	96	1.29	0.24	1.30	1.1	1.4	0.6	1.8	1.13	0.2	1.10	1	1.2	0.1	1.8	< 0.001
Lower right first bicuspid	96	1.06	0.18	1.00	1	1.2	0.7	1.5	1.01	0.16	1.00	0.9	1.1	0.6	1.5	0.012
Lower right second bicuspid	93	1.03	0.2	1.00	0.9	1.1	0.7	1.9	1.12	0.27	1.00	0.9	1.3	0.8	1.9	< 0.003

#### 3.1.1 PETr

Before conducting an IPR procedure, it is essential to estimate the PETr before determining the available amount of IPR for each mesial and distal site, allowing for an effective IPR planning strategy. Typically, the amount of IPR ranges from a minimum of 0.1 mm to a maximum of 0.6 mm for each interproximal site. To estimate the amount of PETr, we considered a single site (mesial or distal) IPR ranging from 0.1 to 0.6 mm. A PETr above 0.5 mm was considered to indicate a safe area and a PETr below 0.3 mm was considered to indicate a high‐risk area. The overall PETr mean for a single site was 0.58 mm (±0.14) for the mesial plane and 0.57 mm (±0.14) if an IPR less than 0.4 mm was planed (Table 2).

**Table 2:** Proximal thickness enamel remaining after considering IPR total per side.

Teeth	0.1 mm of IPR		0.2 mm of IPR		0.3 mm of IPR		0.4 mm of IPR		0.5 mm of IPR		0.6 mm of IPR	
	Mesial PETr	Distal PETr	Mesial PETr	Distal PETr	Mesial PETr	Distal PETr	Mesial PETr	Distal PETr	Mesial PETr	Distal PETr	Mesial PETr	Distal PETr
	Mean	Mean	Mean	Mean	Mean	Mean	Mean	Mean	Mean	Mean	Mean	Mean
UR second bicuspid	1.05	1.01	0.95	0.91	0.85	0.81	0.75	0.71	0.65	0.61	0.55	0.51
UR first bicuspid	0.97	0.95	0.87	0.85	0.77	0.75	0.67	0.65	0.57	0.55	0.47	0.45
UR cuspid	1.08	1.15	0.98	1.05	0.88	0.95	0.78	0.85	0.68	0.75	0.58	0.65
UR lateral incisor	0.95	0.89	0.85	0.79	0.75	0.69	0.65	0.59	0.55	0.49	0.45	0.39
UR central incisor	0.98	1	0.88	0.9	0.78	0.8	0.68	0.7	0.58	0.6	0.48	0.5
UL central incisor	1.07	1.08	0.97	0.98	0.87	0.88	0.77	0.78	0.67	0.68	0.57	0.58
UL lateral incisor	0.96	0.91	0.86	0.81	0.76	0.71	0.66	0.61	0.56	0.51	0.46	0.41
UL cuspid	1.15	1.05	1.05	0.95	0.95	0.85	0.85	0.75	0.75	0.65	0.65	0.55
UL first bicuspid	0.96	0.94	0.86	0.84	0.76	0.74	0.66	0.64	0.56	0.54	0.46	0.44
UL second bicuspid	0.92	0.97	0.82	0.87	0.72	0.77	0.62	0.67	0.52	0.57	0.42	0.47
LL second bicuspid	0.88	0.94	0.78	0.84	0.68	0.74	0.58	0.64	0.48	0.54	0.38	0.44
LL first bicuspid	0.9	0.91	0.8	0.81	0.7	0.71	0.6	0.61	0.5	0.51	0.4	0.41
LL left cuspid	1.12	1	1.02	0.9	0.92	0.8	0.82	0.7	0.72	0.6	0.62	0.5
LL lateral incisor	0.98	0.98	0.88	0.88	0.78	0.78	0.68	0.68	0.58	0.58	0.48	0.48
LL central incisor	1	0.97	0.9	0.87	0.8	0.77	0.7	0.67	0.6	0.57	0.5	0.47
LR central incisor	0.95	0.89	0.85	0.79	0.75	0.69	0.65	0.59	0.55	0.49	0.45	0.39
LR lateral incisor	0.94	0.87	0.84	0.77	0.74	0.67	0.64	0.57	0.54	0.47	0.44	0.37
LR cuspid	1.19	1.03	1.09	0.93	0.99	0.83	0.89	0.73	0.79	0.63	0.69	0.53
LR first bicuspid	0.97	0.91	0.87	0.81	0.77	0.71	0.67	0.61	0.57	0.51	0.47	0.41
LR second bicuspid	0.93	1.02	0.83	0.92	0.73	0.82	0.63	0.72	0.53	0.62	0.43	0.52

Abbreviations: IPR, interproximal reduction; LL, lower left; LR, lower right; PETr, proximal enamel thickness remaining; UR, upper right; UL, upper left.

Our proposed risk classification for PETr is based on the consideration that even a thin layer of proximal enamel thickness provides protection to the inner tissues, as indicated by Sarig.

#### 3.1.2 Threshold Values for PETr

**Figure 2:**
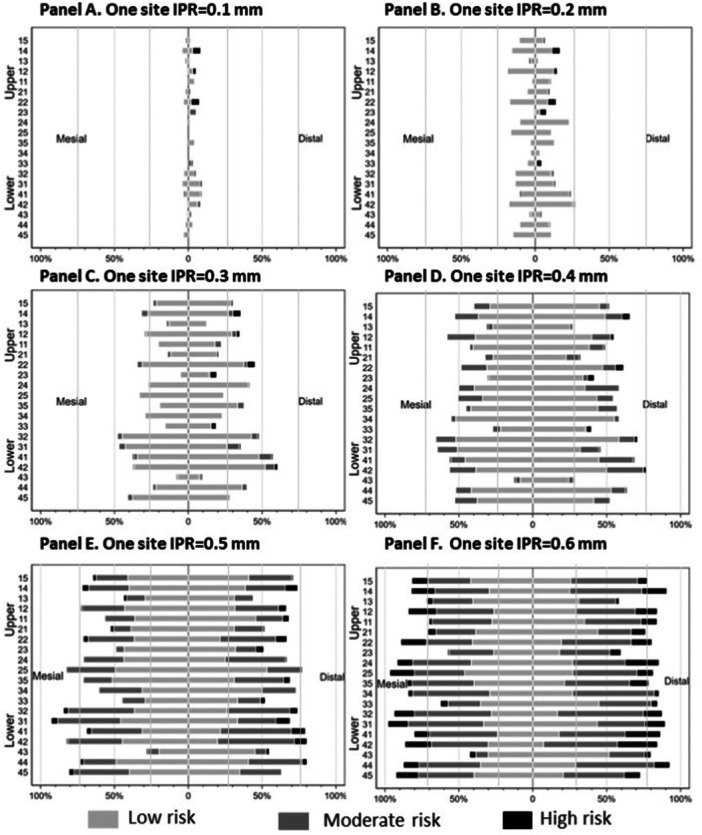
The proximal enamel thickness available (PET‐a) was measured using on‐demand software. Footnote: Details the remaining proximal enamel thickness remaining (PETr) after performing interproximal enamel reduction (IPR) at increments ranging from 0.1 to 0.6 mm per side. The table lists the mean values of mesial and distal enamel thickness across various teeth, demonstrating the progressive reduction in enamel as the extent of IPR increases. In each panel, risk was segregated into three categories, low, moderate, and high, indicated by the absence of color, gray, and black bars, respectively.

Understanding the PETr sites at risk is critical if no previous measurements are performed. Sites were identified as having no risk (> 0.75 mm), low risk (≥ 0.50 mm to < 0.75 mm), moderate risk (≥ 0.30 mm to < 0.50 mm), or high risk (< 0.30 mm) (Figure 2). A single IPR site between 0.1 and 0.2 mm in length was identified, representing a space gain between 0.2 and 0.4 mm for the interproximal site. The overall PETr risk percentage was less than 25%, indicating that fewer than one in four teeth will be at risk if a single 0.2 mm IPR is performed (Figure 2, Panels A and B). Similarly, if a 0.3 mm IPR is performed, the PETr risk percentage increases between 25% and 50% for the following teeth: 15, 14, 12, 22, 24, 32, 31, 41, 42, 44, and 45. The PETr risk notably increased in the lower anterior teeth and represented up to a 0.6 mm space gain per interproximal site (Figure 2, Panel C). For the 0.4–0.6 mm IPR group, almost every tooth presented an increased PETr risk ranging from 50% to 75% and was classified as moderate to high risk (Figure 2, Panels D, E, and F).

##### 3.1.2.1 Summary of the PETa and Maximum IPR Suggested

**Figure 3:**
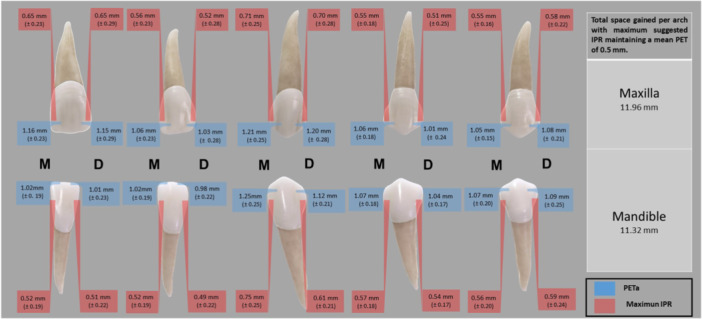
Summary of total space gained per arch using the maximum suggested IPR with a mean PET of 0.5 mm. Footnote: Figure 3 illustrates the total space gained per arch using the maximum suggested interproximal reduction (IPR), ensuring a mean proximal enamel thickness available (PETa) of 0.5 mm per tooth. The blue bars represent the PETa values (mean ± standard deviation) for the mesial (M) and distal (D) surfaces of each tooth, whereas the red bars indicate the maximum IPR that can be performed while maintaining the specified PETa threshold. The total space gained is summarized for the maxilla and mandible.

Figure 3 shows the total space gained per arch through maximum suggested IPR while maintaining a mean proximal enamel thickness (PETa) of 0.5 mm per tooth. For both the maxilla and mandible, the PETa values are consistently higher than the corresponding maximum IPR values, ensuring enamel preservation. The total space gained is 11.96 mm for the maxilla and 11.32 mm for the mandible, highlighting the balance between effective space creation and enamel integrity. This summarizes the clinical feasibility of maintaining a safe PETa threshold and PETr during orthodontic treatments.

## 4 Discussion

Our study focused on the measurement of proximal enamel thickness (PETa) and the estimation of PETr to determine the optimal amount of IPR achievable without increasing the risk of side effects. On the basis of our results, we find that PETa and PETr can serve as reliable indicators for establishing an accurate IPR. Instead of solely estimating the space needed for crowding corrections, we advocate a comprehensive evaluation of the enamel available before determining the effective IPR amount. Measuring PETa and PETr during IPR planning ensures a precise, customized approach while minimizing the risk of excessive enamel reduction.

We observed an overall mean PETa value of 1.0 mm. However, 65% of the mesial sites and 80% of the distal sites showed minimal initial PETa measurements from both the mesial and distal aspects, with values equal to or less than 0.7 mm. These findings highlight the potential limitations of IPR due to insufficient enamel thickness, necessitating careful evaluation during treatment planning to avoid compromising dental integrity.

Mesial and distal proximal enamel thicknesses (PETa) were evaluated in our study. The mean distal PETa values were frequently greater than the mesial values, ranging from 0.96 to 1.25 mm and from 0.98 to 1.0 mm, respectively. Markedly greater differences between the distal and mesial sides in the upper and lower cuspids and seven out of the 20 (35%) teeth (14, 25, 31, 41, 42, 44, and 45) were observed. An inverse relationship was observed in four out of the 20 (20%) teeth. The PETa of the remaining 45% of the teeth did not significantly differ between the mesial and distal sides. These findings are consistent with those of previous studies conducted by Kailasam et al. (2021), Bosio (2022), Harris and Hicks (1998), Vellini‐Ferreira et al. (2012), Sarig et al. (2015), Stroud, Buschang, and Goaz (1994), and Macha et al. (2010), which reported similar ranges of PETa values. However, these differences, when present, are within a close range (0.1 mm) and do not represent clinically significant discrepancies. Therefore, it is essential to measure PETa to account for such differences to avoid excessive enamel reduction, particularly when large distal IPRs are considered.

Our investigation revealed that the overall mean PETr still exceeded 0.5 mm for both the mesial and distal sites when a single‐site IPR of 0.4 mm was planned. However, we caution against single‐site IPRs exceeding 0.20 mm, as they significantly increase the proportion of interproximal sites at moderate (PETr: 0.49–0.3 mm) or high (PETr: < 0.3 mm) risk. This finding indicates that exceeding the recommended threshold may lead to adverse outcomes and complications.

Another relevant finding of our study emphasizes the importance of establishing a maximum limit prior to IPR to ensure safe PET‐r. Although there is a common belief that a maximum wear of 0.5 mm is safe (Meredith et al. 2017), our results indicate that even an IPR of 0.1 mm could result in a PETr below the threshold of 0.3 mm, which carries a higher risk of sensitivity and potential complications (West et al. 2012, 2013). The need for personalized planning of IPR must be based on the available PETa to avoid excessive enamel reduction while minimizing the risks associated with orthodontic treatment.

The use of CBCT measurements to accurately estimate PETa is particularly valuable when planning multiple‐site IPRs exceeding 0.20 mm or single‐site reductions greater than 0.4 mm. Our study is among the first to use in vivo CBCT for measuring PETa. Previous applications of CBCT were reported by Akli et al. (2020) and Bian et al. (2020) and relied on in vitro micro‐CBCT studies. However, micro‐CBCT involves significantly greater radiation exposure. Other studies have used traditional methods such as bitewing radiographs or periapical radiographs (Hall et al. 2007; Harris and Hicks 1998; Stroud, Buschang, and Goaz 1994) and profilometry (Vellini‐Ferreira et al. 2012; Macha et al. 2010; Fernandes et al. 2011), which are prone to errors caused by tooth positioning, head tilting, magnification artifacts, and overlapping structures. CBCT provides high‐resolution, three‐dimensional imaging that minimizes these limitations. This precision enables a more accurate evaluation of enamel thickness across mesial and distal surfaces, facilitating personalized and effective treatment planning.

Additionally, CBCT overcomes the inherent limitations of in vitro methods such as microscopy and microcomputed tomography (micro‐CT) (Hamba et al. 2012), which cannot replicate clinical conditions, making it a practical tool for in vivo applications (Huaiquin‐Zúñiga et al. 2024). Its capacity to generate detailed axial, sagittal, and coronal views enhances diagnostic accuracy, particularly in complex cases involving thin enamel regions or atypical tooth morphology. CBCT‐based PETa measurements before IPR enable precise PETr estimations, even within the 0.30 mm voxel dimensions of the FOV typically used in orthodontics. This approach supports safe enamel preservation while reducing risks such as sensitivity, structural compromise, and overreduction.

This precautionary use of CBCT effectively mitigates potential side effects associated with excessive enamel removal, thereby enhancing patient safety and improving treatment outcomes. Additionally, understanding enamel thickness is critical for veneer preparation to avoid excessive enamel removal and to ensure the longevity of the restoration. Studies have shown that enamel thickness varies significantly across different regions of the tooth, necessitating individualized treatment plans (Pahlevan et al. 2014).

Our results suggest that measuring PETa prior to IPR is a valuable tool for achieving precise and individualized planning in orthodontic treatment. Providing detailed information about the available enamel thickness at each site facilitates accurate estimation of the necessary IPR to achieve safe PETr. This practice improves the predictability of dental movements, ensures the generation of adequate space, and minimizes risks associated with IPR procedures.

However, it is crucial to consider the increased radiation exposure associated with CBCT, which is approximately 3.4–4.7 times greater than that of panoramic radiographs (Omidi et al. 2022; Alshomrani 2024; De Grauwe et al. 2019). CBCT should therefore be used selectively, with careful consideration of its benefits and risks. Alternative imaging options that offer adequate diagnostic information with reduced radiation exposure should also be considered. In this context, it is worth noting that the largest FOV typically used in orthodontic diagnostics can help minimize radiation exposure while maximizing the diagnostic utility of a single tomography session (Alshomrani 2024; De Grauwe et al. 2019; Ghanbarnezhad Farshi et al. 2019; Jadhav, Desai, and Tadinada 2023). The primary objective remains to achieve precise measurements while prioritizing patient safety, a fundamental principle in orthodontic treatment planning and management (Alshomrani 2024; De Grauwe et al. 2019; Jadhav, Desai, and Tadinada 2023).

### 4.1 Expanded Clinical Implications

The findings of this study underscore the significant clinical value of incorporating CBCT scans into orthodontic treatment planning, particularly in procedures involving IPR. CBCT offers accurate and individualized measurements of PETa and PETr, allowing clinicians to account for enamel variability among patients and tooth types. This personalized approach reduces the risk of overreduction, especially in teeth with naturally thinner enamel, thereby supporting more effective and patient‐specific treatment planning.

Furthermore, PETa and PETr measurements serve as critical tools for identifying and avoiding moderate‐ to high‐risk cases, preserving enamel integrity and minimizing complications such as hypersensitivity and increased caries susceptibility. By establishing a 0.20 mm threshold for single‐site IPR, this study highlights the need to avoid excessive reductions, particularly in teeth with PETa values below 0.7 mm, where risks increase.

The results also reveal statistically significant differences in mesial and distal PETa values, especially in cuspids, emphasizing the importance of tailored IPR strategies on the basis of tooth‐specific morphology. This individualized approach enables clinicians to balance enamel reduction while maintaining the structural and functional integrity of each tooth.

Moreover, these findings reinforce the importance of prioritizing enamel preservation while achieving aesthetic and functional orthodontic goals. Using CBCT‐derived PETa data in digital planning enhances intervention predictability and safety, supporting evidence‐based orthodontic care.

Although the use of PETa and PETr measurements is not mandatory for IPR planning, their incorporation provides valuable clinical advantages, particularly when space deficiencies necessitate IPR as a treatment strategy. These assessments improve treatment precision, aid in informed decision‐making, and minimize risks associated with enamel reduction. This approach reflects advancements in digital orthodontics and aligns with evidence‐based practices, ensuring optimized outcomes and patient‐centered care.

### 4.2 Limitations

The primary limitation of this study lies in the inherent biological variability of the study population, which could influence proximal enamel thickness (PETa) measurements. However, it is crucial to highlight that these variations do not exceed 0.20 mm, ensuring the reliability and applicability of the findings. Despite genetic, dietary, or environmental differences, the results offer consistent data that enhance the understanding of proximal enamel behavior and its relevance to IPR. This finding reinforces the clinical relevance of the findings, even within the context of population‐based variability.

Although CBCT measurements may provide valuable information for estimating PETa and optimizing IPR planning, the potential risks associated with radiation exposure should not be overlooked. The CBCT voxel size of 0.20 mm may limit the precision of the measurements. However, it is important to emphasize that this method should be regarded as a diagnostic aid, accompanied by a clinical evaluation to determine PETa. More crucial than the absolute precision of the method is the identification of sites with PETa < 0.30 mm or estimated PETr values that could pose a risk before scheduling an IPR. Such sites should be avoided, distributed across other teeth, or excluded from reduction if possible.

Despite the clinical utility and good reliability of PETa estimation via CBCT, additional in vitro studies are needed to further evaluate the validity of this method. Our study used a randomized design to minimize selection bias, setting it apart from previous nonrandomized studies, as noted by Kailasam et al. (2021).

The concept of PETr is a conceptual variable derived from arithmetic subtraction, assuming 100% effectiveness of the IPR. However, the effectiveness of IPR depends on the method used to achieve enamel reduction. Despite these limitations, it is not ethical to use follow‐up CBCT to evaluate changes in in vivo studies. Nonetheless, PETr conceptualization offers valuable clinical insights, especially in cases of suspected prior IPR, excessive enamel reduction, or anatomical variations.

Another limitation of our study is the use of predefined PETr thresholds on the basis of clinical parameters reported in the literature (Sheridan 1987, 1997; Chudasama and Sheridan 2007; Stroud, Buschang, and Goaz 1994; Sarig et al. 2015; Hudson 1956) rather than on validated classifications. These thresholds, organized and proposed by the authors, provide practical guidance for estimating enamel preservation and managing IPR. Although they are not yet part of a formalized risk classification system, they align with parameters commonly used in daily clinical practice when performing IPR.

Despite the clinical utility of these thresholds, further research is needed to validate these parameters and establish a robust, evidence‐based classification system. Such validation would increase their reliability and facilitate the development of standardized guidelines for safe and effective IPR procedures. This approach ensures a balance between current clinical practice and the ongoing evolution of scientific evidence, ultimately improving orthodontic treatment outcomes.

## 5 Conclusion

This study highlights critical considerations for the clinical application of IPR in orthodontic treatment, emphasizing the importance of precision, individualization, and the integration of advanced technologies. The incorporation of PETa and PETr measurements into treatment protocols provides significant clinical benefits that enhance patient care and optimize treatment outcomes.

## Author Contributions

**Enrique González‐García, Nasib Balut‐Chahin, Luis Pablo Cruz‐Hervert,** and **Jean Marc Retrouvey:** conceptualization and methodology. **Enrique González‐García** and **Luis Pablo Cruz‐Hervert: Resources. Nasib Balut‐Chahin:** supervision. **María Eugenia Jiménez Corona, Luis Pablo Cruz‐Hervert,** and **Jean Marc Retrouvey:** formal analysis. **Claudia Daniela Rojo‐Arce,** and **María Eugenia Jiménez Corona:** investigation. **Claudia Daniela Rojo‐Arce:** verification and data curation. **Enrique González‐García, Nasib Balut‐Chahin, Claudia Daniela Rojo‐Arce, María Eugenia Jiménez Corona, Luis Pablo Cruz‐Hervert,** and **Jean Marc Retrouvey:** writing–original draft preparation. **Nasib Balut‐Chahin, María Eugenia Jiménez Corona, Luis Pablo Cruz‐Hervert,** and **Jean Marc Retrouvey:** writing–review and editing. **María Eugenia Jiménez Corona:** visualization. **Luis Pablo Cruz‐Hervert:** project administration.

## Conflicts of Interest

The authors declare no conflicts of interest.

## Data Availability

The data sets used in the current study can be obtained from the corresponding author upon reasonable request.
